# The complete chloroplast genome of *Epilobium hirsutum* L. (Onagraceae)

**DOI:** 10.1080/23802359.2021.1945968

**Published:** 2021-07-01

**Authors:** Fei Meng, Weimin Jiang, Liping Wu, Jing Zhang, Xiaoyan Yao, Jing Wu, Xiaohu Guo, Shihai Xing

**Affiliations:** a College of Pharmacy, Anhui University of Chinese Medicine, Hefei, China; b College of Life Sciences and Environment, Hengyang Normal University, Hengyang, China; c Institute of Traditional Chinese Medicine Resources Protection and Development, Anhui Academy of Chinese Medicine, Hefei, China; d Anhui Province Key Laboratory of Research & Development of Chinese Medicine, Hefei, China

**Keywords:** *Epilobium hirsutum*, Onagraceae, chloroplast genome, phylogenetic tree

## Abstract

The complete chloroplast genome sequence of *Epilobium hirsutum* L. is presented here. It is 161,111 bp in length and divides into four distinct regions: a small single-copy region (SSC) of 17,310 bp, a large single-copy region (LSC) of 89,117 bp, and a pair of inverted repeat (IR) regions of 27,342 bp. The chloroplast genome of *E. hirsutum* includes a total of 125 genes, consisting of 31 tRNA genes, 8 rRNA genes, and 86 protein-coding genes. A phylogenetic tree was generated to evaluate the evolutionary relationship between *E. hirsutum* and relevant species. The chloroplast genome sequencing and phylogenetic analysis offer genetic background for conservation and phylogenetic studied of this species.

*Epilobium hirsutum* L., which belongs to genus *Epilobium*, the largest genus of the Onagraceae family which consists of approximately 200 species, is widely distributed all over the world (Michael 2010). It can be found in moist wastelands of the Mediterranean region, Europe, Asia, and Africa (Karakurt et al. 2013). The medicinal parts of *E. hirsutum* are the herb and the roots (Somayeh et al. 2012). *E. hirsutum* earlier has been claimed to show antinociceptive, anti-inflammatory, antioxidant, antimicrobial, and antitumor effects (Sheikh et al. 2017). Furthermore, the plant has also shown a promising role in treating enlarged prostate, prostatitis, cystitis, burning sensation in urine, and after the prostate operation (Mykhailenko et al. 2019). Although several phylogenetic studies reported transcriptome sequencing and small RNA sequencing of *E. hirsutum* (Kevin et al. 2011; Tan et al. 2018), the complete chloroplast genome sequence is not available till now. Here, we report the complete chloroplast genome sequence of *E. hirsutum* to provide a genomic resource and to clarify the phylogenetic relationship of this plant with other species in the Onagraceae family and other related plants. The results will help to better understand the phylogenetic position of the species and enlarge its further evolutionary studies. Meanwhile, the study will provide a valuable organelle molecular basis genetic information which is helpful to protect the endangered germplasm and further breeding.

Total genomic DNA was extracted from the fresh and healthy leaves of a single individual of *E. hirsutum* sampled from Anhui University of Traditional Chinese Medicine (N31°56′17.41′′; E117°23′24.04′′). Voucher specimens were deposited in the Center of Herbarium, Anhui University of Traditional Chinese Medicine, Hefei, China (AhtcmH, yxy.ahtcm.edu.cn/info/1006/6713.htm, Shi-hai Xing, xshshihai@163.com, under the voucher number 20210319). The total genomic DNA was extracted from the above leaves by a commercial DNAsecure Plant Kit (TIANGEN Biotech Co., Ltd., Beijing, China). The quality and integrity of DNA were checked by BioPhotometer Plus (Nucleic acid and protein detector, Eppendorf, Germany), and 1% agarose gels, and high quality of DNA was used to construct the library. VAHTS™ Universal DNA Library Prep Kit for Illumina^®^ V3 (Vazyme Biotech Co., Ltd., Nanjing, China) was used for DNA library construction and the template size is from 420 bp to 520 bp, and the main peak of our library is within the range. Sequencing was performed by Genewiz Biotechnology Co. Ltd. (Suzhou, China). The isolated genomic was manufactured to average 500 bp paired-end (PE) library using Illumina Hiseq platform (Illumina, San Diego CA, USA), and sequenced by using a 2 × 150 paired-end (PE) configuration. The chloroplast genome was assembled using the program NOVOPlasty 2.7.2 (Dierckxsens et al. 2017), with the complete chloroplast genome of *E. ulleungensis* the reference (GenBank accession no. is NC039575). The draft sequence obtained was corrected manually by clean-read mapping using bowtie2 (Langmead and Salzberg 2012) and Tablet (Milne et al. 2013). Software of prodigal (version 3.02) (Hyatt et al. 2010) was used for gene prediction, and databases of NR (Non-Reduction Protein Database), KEGG (Kyoto Encyclopedia of Genes and Genomes), GO (Gene Ontology Consortium) were used for genome annotation. The gene function annotation in this study is by comparing the amino acid sequences of the predicted coding gene with the protein contained in each of the aforementioned databases, the blast software (version 2.2.31 +) was used to compare with the protein sequence in the database. The value of sequence alignment was set to 1e − 5, and the length of sequence alignment should not be less than 60% of the protein length. If the protein sequence of a gene has significant sequence similarity with the protein sequence in the database, it can be inferred that the gene has the same or similar function as the protein in the database. The best matching result was selected as the annotation result of the gene.

The complete chloroplast genome of *E. hirsutum* was a circular form of 161,111 bp in length, which was separated into four distinct regions such as a large single-copy region of 89,117 bp, a small single-copy region of 17,310 bp, and a pair of inverted repeat regions of 27,342 bp respectively. Overall, GC content of chloroplast genomes is 38.09%, while the value of the LSC, SSC, and IR regions are 36.22, 33.09, and 42.74%, respectively. The chloroplast genome contained a total of 125 genes including 86 protein-coding genes, 31 tRNA genes, and 8 rRNA genes.

To help us understand the phylogenetic position of *E. hirsutum* in family Onagraceae, *E. hirsutum* and other 24 species sequences were used to reconstruct the phylogenetic tree. The complete chloroplast genomes were aligned by MAFFT v7 (Katoh and Standley 2013). Then, the evolutionary history was inferred by using the maximum-likelihood (ML) approach in MEGA7.0 (Kumar et al. 2016) in the Tamura–Nei substitution model (Kumar et al. 2018) and with 1000 bootstrap replicates and settings as described by Stamatakis et al. (2008) (Figure 1). The result of phylogenetic analysis based on 25 chloroplast genomes revealed that *E. Hirsutum* is clustered with *Epilobium ulleungensis* J. M. Chung (They are both in genus Epilobium) in a clade in Onagraceae. This published complete *E. hirsutum* chloroplast genome will provide useful information for the relationships among the major lines of angiosperms.

**Figure 1.**
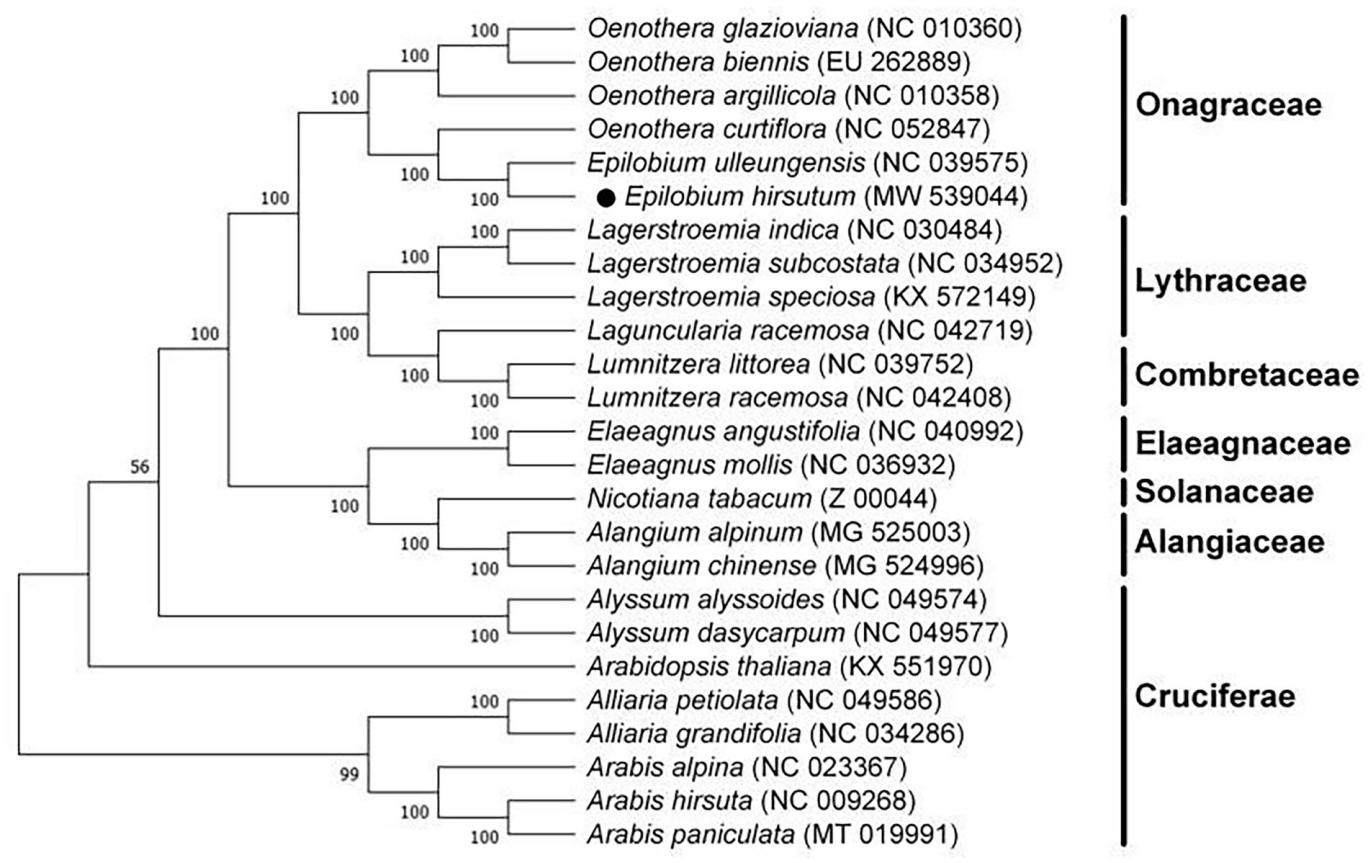
Phylogenetic tree plotting using maximum-likelihood method based on an alignment of the completed chloroplast genome sequences of *Epilobium hirsutum* L. and 24 other representative species. The bootstrap parameter was set as 1000 replicates.

## Data Availability

The genome sequence data that support the findings of this study are openly available in GenBank of NCBI (https://www.ncbi.nlm.nih.gov/nuccore/MW539044) under the access number MW539044.1. The associated BioProject, SRA, and Bio-Sample numbers are PRJNA734095, SSR16695909, and SAMN19471490 respectively.
